# Platelet-Rich Fibrin with ***β***-Tricalcium Phosphate—A Noval Approach for Bone Augmentation in Chronic Periapical Lesion: A Case Report

**DOI:** 10.1155/2012/902858

**Published:** 2012-10-21

**Authors:** K. B. Jayalakshmi, Shipra Agarwal, M. P. Singh, B. T. Vishwanath, Akash Krishna, Rohit Agrawal

**Affiliations:** ^1^Department of Conservative Dentistry and Endodontics, Peoples College of Dental Sciences & Research Centre, Bhopal 462037, India; ^2^Century Dental College, Kerala, Poinachi 671541, India

## Abstract

*Introduction*. This paper describes a case of bone augmentation with combination of Platelet-Rich Fibrin (PRF) and **β**-TCP for treatment of chronic periapical cyst. The case was followed for 12 months. *Methods*. Patient presented with chronic periapical lesion in maxillary anterior teeth with history of trauma 8 years back. Radiographically, a periapical cyst was seen in relation to maxillary left central and lateral incisors. Conventional endodontic treatment was started. Since it was not successful, apical surgery was performed. Bone augmentation was done using PRF in combination with **β**-TCP bone graft to achieve faster healing of the periapical region. Regular followups at 3, 6, 9, and 12 months were done. *Results*. Healing was uneventful. Follow-up examinations revealed progressive, significant, and predictable clinical and radiographic bone regeneration/healing without any clinical symptoms. *Conclusions*. Combined use of PRF and **β**-TCP for bone augmentation in treatment of periapical defects is a potential treatment alternative for faster healing than using these biomaterials alone.

## 1. Introduction

Bacterial infection of the dental pulp may lead to periapical lesions [1]. They are generally diagnosed either during routine dental radiographic examination or following acute pain and/or swelling in relation to the affected tooth [2]. Most periapical lesions (>90%) can be classified as dental granulomas, abscesses, or radicular cysts [3, 4]. The incidence of cysts within periapical lesions varies between 6 and 55% [5]. The occurrence of periapical granulomas ranges between 9.3 and 87.1%, and of abscesses between 28.7 and 70.07% [6].

The ultimate goal of endodontic therapy is to return the involved tooth to a state of health and function [7]. All inflammatory periapical lesions should be initially treated with conventional endodontic therapy [8] which has shown success in 85% of cases [9–11]. However failure after conventional root canal treatment calls for surgical intervention [12]. Periapical Surgery has many limitations, like it is an invasive procedure, has psychological impact on the patient, and requires skilled and experienced operator [13, 14]. Nevertheless, periapical surgery remains the last resort when root canal treatment either fails or is not possible.

Traditional surgical approaches to treat periapical defects include debridement of apical lesions along with reshaping of the surrounding bone, resection, and retro filling of root apex, where healing is almost always by repair [15]. Repair is defined as the healing of a wound by tissue that does not fully restore the architecture or the function of the part [16]. Since this is not ideal, newer approaches such as regenerative procedures that aim to restore lost tissue have been introduced.

Beta-tricalcium phosphate (*β*-TCP) is an alloplast widely used in periapical surgery to enhance new bone formation. It is an osteoconductive bone graft which gets chemically resorbed with a concomitant release of bioactive ions [15].

More number of platelets deliver an increased number of polypeptide growth factors that regulate cell proliferation, chemotaxis, and differentiation to the surgical area [17]. Platelet rich plasma (PRP), first generation of autologous platelet concentrate, has been used for the purpose of tissue regeneration [17, 18]. Although its use has shown clinical success its complex preparation protocol and moderate benefits limit its usage in regenerative surgeries [19, 20].

Platelet rich fibrin (PRF), introduced by Choukroun et al. in the year 2001, is a second-generation platelet concentrate enriched with platelets and growth factors which promote periapical tissue regeneration and healing. Unlike PRP, it is obtained from a anticoagulant and thrombin free blood harvest making it free from the risk of disease transmission [20].

PRP has been successfully used with bone grafts like *β*-TCP for bone regeneration in the treatment of periodontal defects [17, 21].

In the present case an innovative idea of combining PRF with a *β*-tricalcium phosphate was used. Indeed, separate studies have shown clinical success in bone formation with the use of both these materials used separately. This case report presents an attempt to evaluate the healing kinetics of the combination of PRF and *β*-tricalcium phosphate as opposed to using these materials alone.

## 2. Case Report

**Figure 1:**
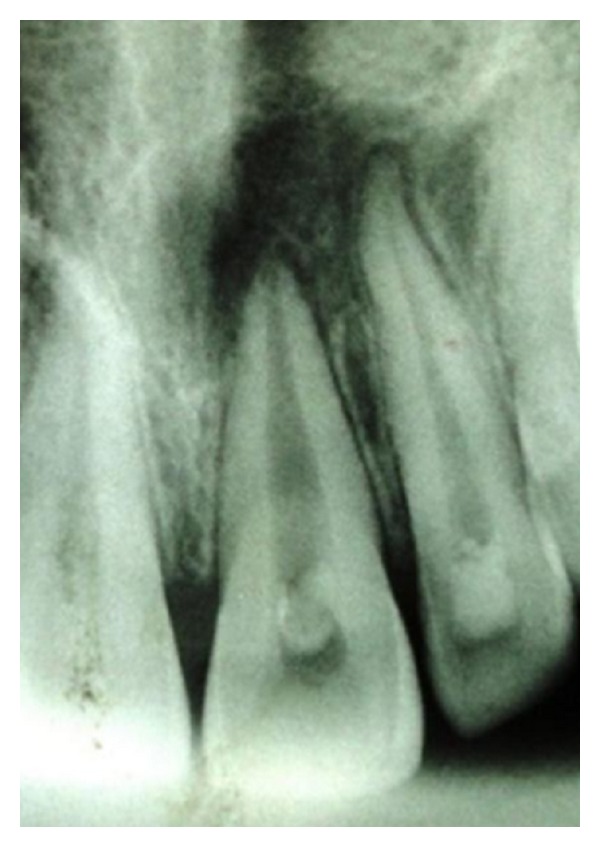
Preoperative radiograph of 21 and 22 showing a large periapical radiolucency.

A 25-year-old female reported to the Department of Conservative Dentistry and Endodontics with chief complaint of swelling and pus discharge from upper front tooth region since 1 month. Past dental history revealed trauma which she sustained 8 yrs back in the same region. On intraoral examination, there was a draining sinus, in relation to the apex of 21. On Electric pulp testing, tooth number 22 was also found nonvital. Periapical radiograph revealed a large diffused periapical radiolucency in relation to 21 and 22 measuring 1.4 cm in diameter (Figure 1).

### 2.1. Management

Culture and sensitivity test revealed presence of *Pseudomonas aeruginosa*. Accordingly an antibiotic course of cefixime 400 mg twice daily and metronidazole 200 mg thrice daily were advised to the patient for 7 days.

Conventional RCT was started with 21 and 22. Since it was not successful it was decided to surgically debride the lesion, with root resection followed by retrograde restoration. In order to achieve optimal healing and regeneration of bone, it was planned to use PRF in combination with bone graft. An ethical clearance was obtained from the institutional ethical committee. Patient consent was taken after careful explanation of the surgical procedure used and the risks and benefits.

Before the surgery, patient's complete hemogram was done and all the parameters were found to be within normal limits.

**Figure 2:**
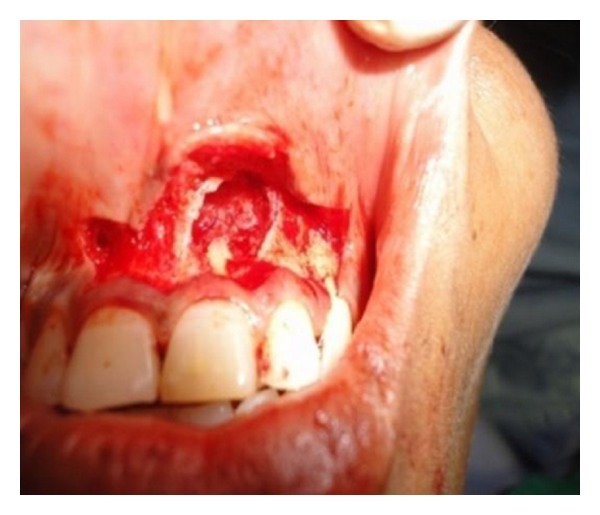
Periapical defect after flap reflection.

Intraoral and extraoral antisepsis was performed using 0.2% chlorhexidine digluconate rinse and povidone iodine solution, respectively. Following administration of local anaesthesia, submarginal incision was given 3 mm apical the marginal gingiva and mucoperiosteal flap was reflected (Figure 2). Meticulous defect debridement was done; 21 and 22 were then obturated using lateral and vertical condensation technique.

**Figure 3:**
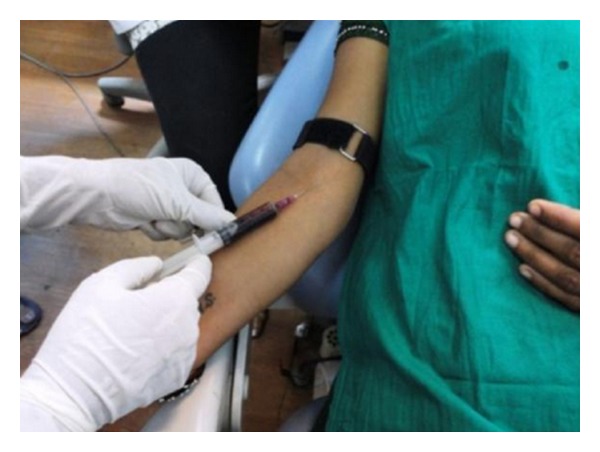
Blood collection from anticubital vein.

**Figure 4:**
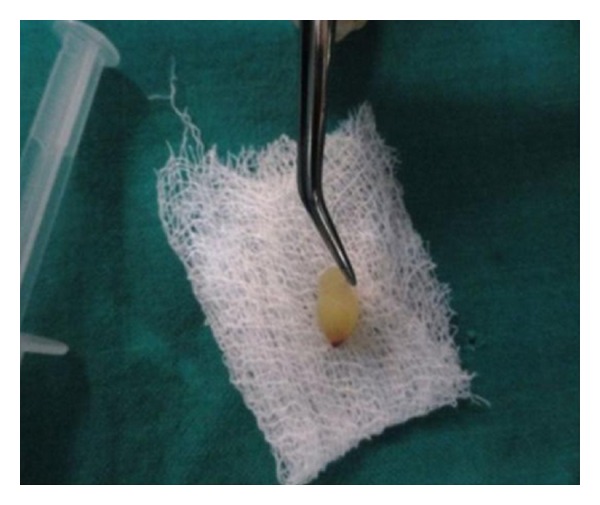
PRF clot obtained after centrifugation.

PRF was prepared in accordance with the protocol developed by Freymiller and Aghaloo [19]. Intravenous blood (by venipuncturing of the antecubital vein) was collected (Figure 3) in a 10 mL sterile tube without anticoagulant and immediately centrifuged at 3,000 rpm for 10 minutes. Blood centrifugation allowed the formation of a structured fibrin clot in the middle of the tube, just between the red corpuscles at the bottom and acellular plasma (platelet-poor plasma) at the top. PRF was easily separated from red corpuscles base (preserving a small RBC layer) using sterile tweezers (Figure 4) just after removal of PPP (platelet-poor plasma) and then transferred into a sterile dappen dish.

**Figure 5:**
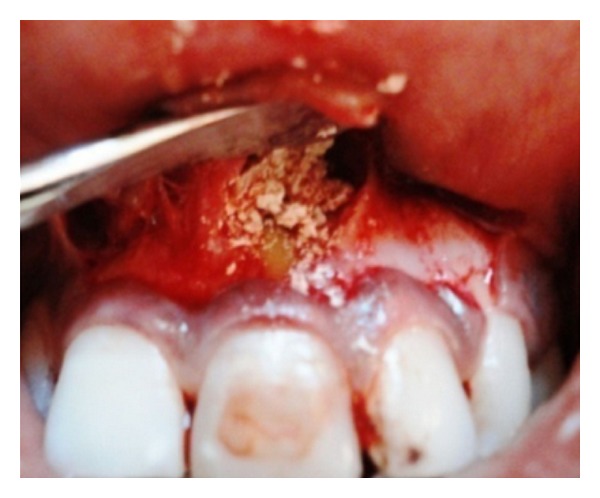
PRF mixed with *β*-tricalcium phosphate placed into the defect.

PRF was mixed with *β*-tricalcium phosphate and augmented into the intrabony defect upto the surrounding bone level (Figure 5). The mucoperiosteal flap was repositioned and simple interrupted sutures were given using 3–0 nonabsorbable black silk suture.

Post-operative care was explained to the patient, with instructions to report back after a week for suture removal. Recall examinations after 3-, 6-, 9-, and 12-month interval were done to evaluate the healing kinetics of the periapical defect.

## 3. Discussion

Regeneration is defined as reproduction or reconstitution of a lost or injured part which fully restores the architecture or function of the part [16].

Regeneration of tissue after periapical surgery requires (a) recruitment of progenitor/stem cells to differentiate into committed cells, (b) growth/differentiation factors as necessary signals for attachment, migration, proliferation and differentiation of cells, and (c) local-microenvironmental cues like adhesion molecules, extra cellular matrix, associated non-collagenous protein molecules, and so forth. Lack of any of these elements would result in repair rather than regeneration [22].

Perhaps the most commonly used technique for regeneration is the use of bone replacement grafts. These grafts can promote tissue or bone regeneration through variety of mechanisms.

Bone grafting materials include autografts, allograft, xenografts, and alloplasts. Alloplasts such as osteoconductive calcium phosphate have been widely used in periapical surgery to enhance new bone formation [15]. Several case reports have demonstrated healing with mature bone and haemopoietic marrow in periapical areas by using this bone graft [23–25].

To promote periapical tissue regeneration and healing, local application of growth factors and host modulating agents is being used to maximize the body's healing potential. TGF-beta and PDGF are the typical two growth factors which promote healing of soft tissue and bone through stimulation of collagen production to improve wound strength and initiation of callus formation [15]. PDGF is aregulator for migration, proliferation, and survival of mesenchymal cell lineages. TGF-beta constitutes the most powerful fibrosing agent among all cytokines. It induces massive synthesis of matrix molecules such as collagen-I and fibronectin either by osteoblasts or fibroblasts. Although its regulation mechanism is particularly complex, it is considered as an inflammation regulator through its capacity to induce fibrous cicatrization. Basic studies have demonstrated that specialized secretory granules of platelets, such as alpha-granules, contain these growth factors [26, 27]. Growth factors are known to attract stem cells present in apical tissues [28].

Platelet-rich plasma (PRP) has been used clinically to stimulate bone regeneration although its real efficacy is debated [17]. It has been suggested to mediate only the early aspects of bone regeneration [29]. Its long-term predictability remains questionable, and the anticipated benefits are moderate [29].

PRF helps to obtain fibrin membranes enriched with platelets and growth factors. PRF by Choukroun's technique is produced in a natural manner, without using an anticoagulant, bovine thrombin, or calcium chloride for platelet activation and fibrin polymerization [20].

In vitro studies have proved that PRF releases autologous growth factors gradually for at least 1 week and up to 28 days [30]. The natural and slow polymerization occurring during centrifugation process of PRF leads to formation of a homogenous 3-dimensional organization of the fibrin network. The absence of anticoagulant in the test tube leads to massive platelet activation, bolstered by the presence of a mineral phase on the tube walls (residual glass particles). A progressive polymerization mode signifies increased incorporation of the circulating cytokines in the fibrin meshes (intrinsic cytokines). This configuration increases the lifespan of these cytokines, as they are released and used only at the time of initial cicatricial remodeling [31]. PRF has a stronger and more durable effect than PRP [20].

Marx et al. in their study added PRP to bone grafts used in mandibular bone defects and evidenced that radiographically the maturation rate was better than that of grafts without platelet-rich plasma [17]. Wiltfang et al. reported 8% to 10% more bone formation when PRP was added to tricalcium phosphate [32].

In another study Goyal et al. compared the healing responses of PRP with guided tissue regeneration Membrane and found significant healing in the treatment of apicomarginal defects [18]. In addition Taschieri et al. also used combination of autologous growth factors with xenogenic bone grafts in treatment of through and through bone lesions and observed a fast and predictable tissue healing [21].

The healing potential of PRF combined with *β*-TCP has not been studied in endodontics. Kim et al. combined PRF with *β*-TCP and observed rapid bone formation, remodeling, and calcification in the second week than the *β*-TCP alone in rabbits [33].

**Figure 6:**
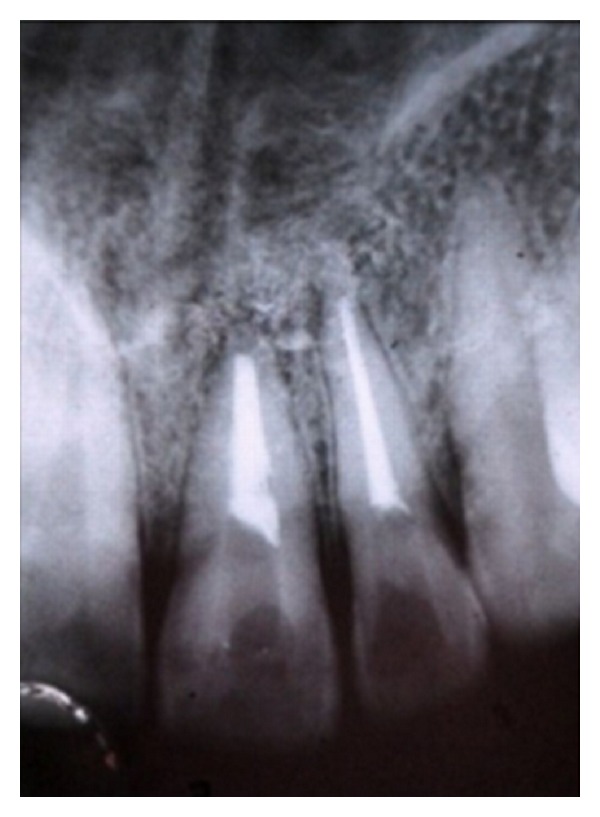
Postoperative radiograph after 3 months.

**Figure 7:**
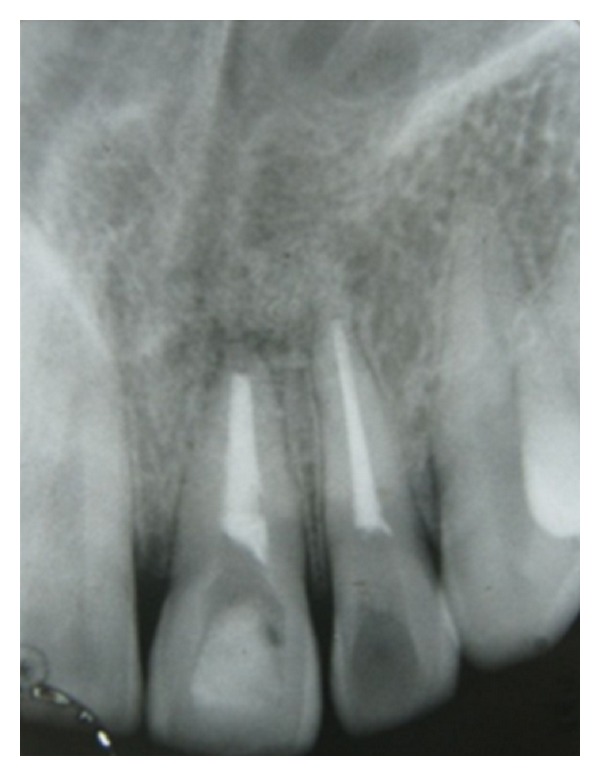
Follow-up radiograph after 6 months (with intracoronal bleaching agent).

**Figure 8:**
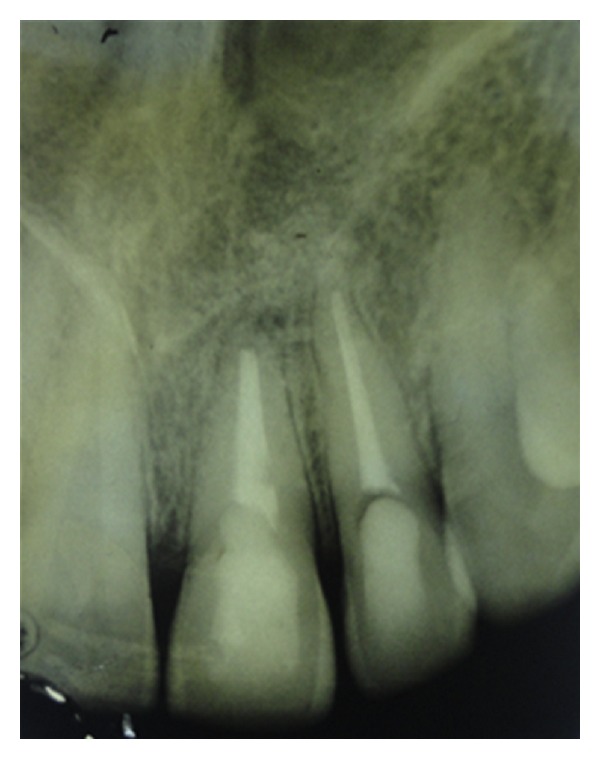
Follow-up radiograph after 9 months.

**Figure 9:**
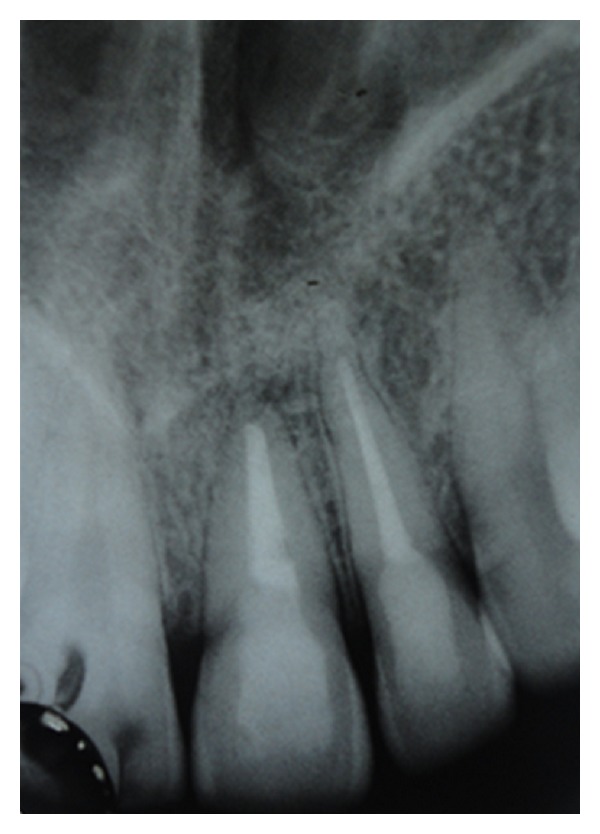
Follow-up radiograph after 12 months.

In the present case, it was observed that at 3- (Figure 6), 6- (Figure 7), 9- (Figure 8), and 12- (Figure 9) month followup after the surgical treatment of large chronic periapical lesion, PRF combined with beta-tricalcium resulted in significant, progressive, and predictable clinical and radiographic bone regeneration.

Besides promoting wound healing, bone growth, and maturation, PRF mixed with *β*-tricalcium phosphate bone graft has the advantages of graft stabilization, wound sealing, hemostasis, and improved handling properties [34].

However, like other clinical studies this study also has few limitations like short follow-up period of 12 months and a need for histological evaluation to confirm regeneration.

## 4. Conclusion

From the present case report, where PRF and *β*-Tricalcium Phosphate allograft were used for periapical healing, following conclusions can be drawn.

- Addition of PRF to *β*-Tricalcium Phosphate allograft accelerates regenerative capacity of bone.
- When used in combination, they give a predictable clinical and radiographic evidence of bone formation.
